# Environmental Regulation, Rural Residents’ Health Investment, and Agricultural Eco-Efficiency: An Empirical Analysis Based on 31 Chinese Provinces

**DOI:** 10.3390/ijerph19053125

**Published:** 2022-03-07

**Authors:** Kun Zhou, Xingqiang Zheng, Yan Long, Jin Wu, Jianqiang Li

**Affiliations:** 1 College of Management, Sichuan Agricultural University, Chengdu 611130, China; zhoukun@stu.sicau.edu.cn (K.Z.); zxq2096115807@163.com (X.Z.); 2 Biogas Institute of Ministry of Agriculture and Rural Affairs, Chengdu 610041, China; longyan01@caas.cn (Y.L.); wujin@caas.cn (J.W.)

**Keywords:** *ER*, *AEE*, RRHI, mediating effects, heterogeneity analysis

## Abstract

This paper explores the effects of environmental regulation (*ER*) and rural residents’ health investment (RRHI) on agricultural eco-efficiency (*AEE*) to provide a reference for the Chinese Government and other developing countries for implementing environmental regulation policies and to provide new paths to further improve green development in agriculture. Using the panel data of 31 Chinese provinces from 2009–2018, the Super-SBM model was used to measure *AEE*. The role of *ER* on *AEE* was analyzed based on panel two-way fixed effects with endogeneity treatment and a robustness test, and this mediating effect analysis was conducted to analyze the role of RRHI in *ER* and *AEE*, examining the extent of the effect of *ER* on *AEE* in three regions of China—eastern, central and western—using a heterogeneity analysis. The results of the study show that: (1) from a national perspective, *ER* has a significant positive impact on *AEE*, showing that *ER* is effective at this stage; (2) when RRHI is used as a mediating variable, the rising *ER*’s intensity can promote *AEE* by increasing RRHI; and (3) the results of the heterogeneity analysis show that *ER* has the greatest impact on *AEE* in the economically developed eastern region; the western region with a weaker level of economic development is in second place. However, *ER* has a negative impact on *AEE* in the central region with a medium level of economic development. Thus, the impact of *ER* on *AEE* will show great differences depending on the stage of economic development.

## 1. Introduction

Since its reform and opening-up policies, China’s industrialization has accelerated significantly, and its socioeconomic development has reached a new level, but the problem of environmental pollution has become increasingly serious, which severely affects agricultural production [1]. For a long time, in order to solve the problem of subsistence, Chinese farmers have used chemical fertilizers and pesticides in large quantities to improve grain production, but at the same time, the excessive use of chemical fertilizers and pesticides has brought serious damages [2], which led to environmental pollution and a series of soil quality degradation problems [3]. According to the *2019 National Arable Land Quality Grade Bulletin* issued by the Chinese Ministry of Agriculture and Rural Development in 2019, more than 20% of China’s arable land is grade 7 to 10; this part of the arable land has a relatively poor basic land quality and significant obstacles to agricultural production, and it is difficult to make fundamental improvements in a short period of time. Additionally, soil pollution caused by excessive heavy metals and the excessive use of chemical fertilizers and pesticides has degraded the quality of arable land to some extent, and the issue of agricultural food safety was once a hot topic of concern for many people. Improving the level opening-up of green agricultural development is the key to solving the problems of resource scarcity and environmental degradation in Chinese agriculture [4]. To actively explore the path of high-yield, ecological and safe agricultural development, the Chinese Ministry of Agriculture and Rural Affairs issued the *Action Plan for Zero Growth of Chemical Fertilizer Use by 2020* and the *Action Plan for Zero Growth of Pesticide Use by 2020* in 2015 to slow down the excessive input of chemical fertilizers and pesticides, and the *Document NO.1 of the Central Government* has also proposed for many years that agricultural green development should move in the main direction of the current development of Chinese agriculture.

Agricultural eco-efficiency (*AEE*) is an important indicator of the level of green agricultural development. In the existing studies on *AEE*, most scholars take agriculture (plantation) as the research object and the number of chemical fertilizers, pesticides, and agricultural films used in the production process as the main evaluation index of *AEE*, adopting the life-cycle method, stochastic frontier production function, or a data envelopment analysis to measure *AEE* as a measure of the level of *AEE* in a country or region [5,6,7,8]. Similarly, a few scholars evaluated the green production behavior preferences of farmers in typical regions and their influencing factors by establishing an agricultural eco-efficiency index system and conducting field research [4,6,9,10]. This paper intends to measure the level of *AEE* in each province of China by using rural residential health investment as a mediating variable to observe the mechanisms of action between *ER*, RRHI and *AEE*, but it is difficult to obtain real-time data on each farmer’s agricultural production factor inputs by using a micro-survey on a national scale. Instead, *AEE* aims to measure the number of chemicals, such as chemical fertilizers, pesticides, and agricultural films that produce certain hazards to crops, soil, and food in the production of farmers; the lower the number of inputs, such as chemical fertilizers and their residues, the lower the degree of hazards to the soil, water systems and food, and the higher the degree of *AEE*. Therefore, macro panel data were used to measure the degree of green production in terms of the residues of fertilizers, pesticides, and agricultural films invested in the production process by farmers and carbon emissions as non-desired outputs.

Environmental regulation (*ER*) refers to the fact that, because environmental pollution has negative externalities, the Government regulates economic activities by formulating the corresponding policies and measures to achieve the goal of harmonizing environmental protection and economic development. Environmental pollution is a hot spot of international concern, and *ER* is a favorable measure for the Government to solve the externality harm caused by environmental pollution. Whether *ER* can effectively promote *AEE* also becomes key for the accurate implementation of national policies. The impact of *ER* on the green development of agriculture is an important judgment basis for measuring the effectiveness of government policy implementation and social governance behavior. Rural residents’ health investment (RRHI) refers to rural residents’ investment in health services (e.g., health care and medical services) to cope with their own diseases and major public health events [11], and it is an important way to improve human capital. RRHI also increases or decreases with changes in environmental conditions, and correspondingly, the physical health status of rural residents plays an important role in agricultural production. Therefore, an in-depth analysis of the mechanisms and effects among *ER*, RRHI and *AEE* is of great significance for improving rural residents’ health and promoting agricultural production. Current scholarly research hotspots in *ER* focus on the role of policies in promoting the green development of the economy [12] and the effect on the structural upgrading of manufacturing or industrial industries [13,14,15,16]. Some scholars have also focused on the economic impact of *ER* in green technology innovation [17,18], ecological improvement [19], and total factor productivity (TFP) [20]. Additionally, in the study of eco-efficiency in the agricultural sector, some scholars focus on the policy combing of *ER* in agriculture [21], the impact of *ER* on the green transformation of agriculture [22], and the green production behavior of farmers [23], the agricultural sector and farm eco-efficiency [24,25,26,27]. However, few scholars have studied the mechanism of the impact of *ER* on *AEE* under different degrees of economic development, and the mediating role of RRHI in *ER* and *AEE*. Therefore, exploring the impact of *ER* on *AEE* under different levels of economic development, and whether *AEE* can be improved by promoting RRHI, is important for the Chinese Government to tailor its policies to local conditions, as well as for rural residents’ livelihood and agricultural sustainable development in various regions of China.

This paper uses macro panel statistics from 2009 to 2018 in 31 provinces of China to analyze the impact and mechanisms of *ER* and RRHI on *AEE* and further employs a heterogeneity analysis to evaluate the impact of *ER* on *AEE* in different regions of economic development. The possible innovations of this paper are the following. First, this article simultaneously incorporates surface source pollution from fertilizers, pesticides and agricultural films and carbon dioxide emissions that may be generated during agricultural production into the *AEE* index system to measure *AEE* from the perspective of carbon emissions, which is not common in the existing relevant literature. Second, on the basis of analyzing whether *ER* can effectively promote *AEE*, the mechanism of the role of RRHI in *ER* on *AEE* is further explored, so as to discover whether RRHI can act as a mediating variable in it and to provide a more rigorous explanation of the role of *ER* on *AEE*. Third, according to its economic development, China is categorized and analyzed according to its economic development status to find out whether *ER*s have different effects on *AEE* in different stages of economic development, which will also provide some theoretical basis for the degree of *ER* implemented to promote *AEE* in countries or regions in the process of economic development.

## 2. Research Mechanism and Hypothesis

### 2.1. The Role of ER on AEE

Any economic activity is a rational activity that maximizes utility under certain institutional constraints, i.e., economic activity is strongly influenced by institutional factors. In agricultural production, environmental regulation (*ER*) refers to a series of guidance, incentives, and constraint measures taken by the Government to promote farmers’ participation in green production and alleviate rural environmental pollution problems [28]. *ER* in agriculture contains three dimensions: guided environmental regulation (GER), incentive-based environmental regulation (IBER), and constraint environmental regulations (CER) [29]. Among them, guided environmental regulation (GER) reduces agricultural environmental pollution by guiding farmers to establish green production concepts and technology; IBER usually refers to a series of economic compensation or incentives implemented by the government to motivate farmers to adopt green fertilization behaviors; CER refers to a series of penalty-based policies and measures introduced by the government to restrain farmers’ non-green production behaviors. *ER* is an important measure to reduce environmental pollution and improve the way of *AEE*. In general, *ER* can influence farmers’ perceived benefits through guiding farmers’ perceptions, financial subsidies, and penalties, which can help promote farmers’ adoption of organic fertilizer behaviors and reduce chemical inputs such as fertilizers and pesticides in agricultural production [28], thus promoting *AEE*. Therefore, this paper proposes the following hypothesis:

**Hypothesis** **1** **(H1).** *ER will enhance the level of AEE.*

### 2.2. ER, RRHI, and AEE

Environmental pollution is an important cause of health hazards for the population [30], and people will increase their health care expenditures when pollution poses a risk to human health. For example, Jerrett et al. (2003) examined the relationship between environmental quality and health care expenditures using cross-sectional data and found that health care expenditures were also higher in areas with high environmental pollution after controlling for other variables that may affect health expenditures [31]. The health of residents is affected not only by environmental pollution and environmental impacts in the region, but also by the total amount of pollutant emissions from surrounding areas and the intensity of *ER*. The improvement in environmental quality is usually seen as a short-term effect of the implementation of regulatory policies, while the improvement in residents’ physical and mental health is seen as a long-term benign effect of sustained environmental quality improvement [32]. The higher intensity of *ER* indicates that the local environmental pollution poses a greater threat to the life and health levels of the residents, and the Government will implement strong *ER* to mitigate the harm caused by pollution to maintain stable social development. For example, to curb the serious damage to air quality caused by pollution emissions from power stations, the Chinese Government enacted a strict pollution emission control decree (*Two Control Zone policy*, TCZ) for power stations in 1998. Based on this, Tanaka (2015) analyzed the impact of this air pollution emission regulation policy on the health of Chinese residents using the double-difference method (DID) [33]. They found that this policy (TCZ) improved the health status of the population and reduced infant and child mortality, while the health expenditures of the population increased significantly. In other words, *ER* rises with the level of pollution, and the rise in *ER* and pollution triggers the population to pay attention to health and make health investments to ensure sufficient production levels. For a long time, Chinese agriculture has required large amounts of labor and land for its development, and good health levels are necessary to carry out agricultural production. As environmental quality declines and the intensity of *ER*s becomes higher, it also raises rural residents’ concern about their health, and they will increase investment in their health to mitigate the health risks associated with environmental pollution and ensuring the physical demands of agricultural production. Therefore, this paper proposes the following hypothesis.

**Hypothesis** **2** **(H2).** *ER can effectively promote the increase of RRHI.*

Human capital is composed of human knowledge, skills, and health qualities, which have economic value and are a combination of the mental and physical qualities of workers [34]. RRHI can promote their own health and improve their human capital, so that they can better adapt to physical and mental activities. Having a healthy body allows for a higher production demand and sustains long-term economic growth. Environmental quality can affect both individual health and productivity levels [35]. Without deliberate intervention, environmental pollution will increase human morbidity and mortality, reduce economic productivity, and impair human capital formation [36]. In the agricultural sector, *AEE* cannot be achieved without the input of high-quality human capital, and the quantity and quality of human capital will have a significant impact on the high-quality development of the agricultural economy. The current development situation in China determines that a large amount of human capital input is required for agricultural production, and the better the health of the workforce and higher the condition of human capital, the more likely it is to meet the requirements of high-intensity *ER*s and promote *AEE* [37]. Therefore, this paper proposes the following hypothesis.

**Hypothesis** **3** **(H3).** *Increasing investment in the health of rural residents can effectively promote AEE.*

**Figure 1:**
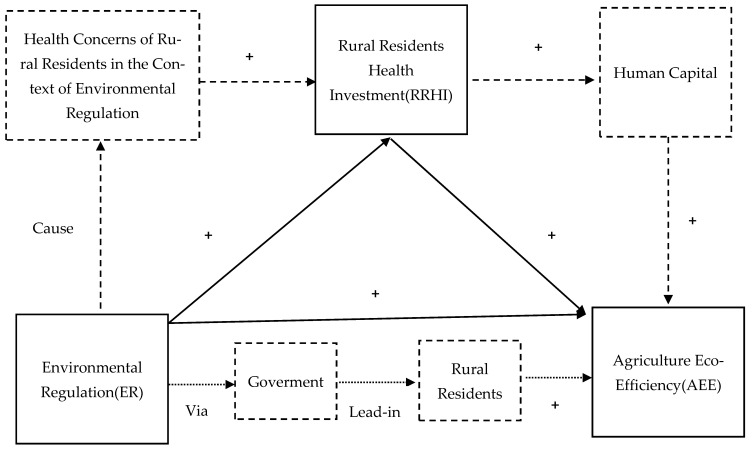
Mechanism analysis framework of *ER*, RRHI, and *AEE*.

If all the above hypotheses are valid, that is, both *ER* and rural residents’ RRHI can promote *AEE* and *ER* can promote RRHI, it can be inferred that RRHI plays a mediating role in the effect of *ER* on *AEE*; therefore, this paper proposes the following hypothesis. Please see Figure 1 for the specific research mechanism in this paper.

**Hypothesis** **4** **(H4).** *RRHI plays a mediating role in the effect of ER on AEE.*

## 3. Research Methodology and Data Sources

### 3.1. Model Construction

#### 3.1.1. Super-SBM Model Based on the Undesired Output

The basic data envelopment model (DEA) model idea is to consider the research object as each decision unit, analyze the input–output ratios of each decision unit comprehensively, operate the input–output weights of each decision unit as variables, and judge whether each decision unit reaches the efficient state through the operation results [38]. The traditional data envelopment model (DEA) does not consider the input–output slackness, and the calculation results have certain errors; therefore, Tone improved the DEA model on this basis and defined the new model as the Super-SBM model [39,40]. The Super-SBM model is a non-radial, non-rectangular model that incorporates slack variables into the model and provides a more accurate measure of efficiency. Therefore, the following Super-SBM model is constructed in this paper:

(1) ρ*=min1m∑i=1mXi−Xi01S1+S2(∑r=1s1Yr−gYr0g+∑t=1s2Yt−bYt0b  

$$s.t.X^{-}\geq \sum_{j=1,\neq 0}^{n}\beta_{j}X_{j},Y^{-g}\leq \sum_{j=1,\neq 0}^{n}\beta_{j}Y_{j}^{g},Y^{-b}\leq \sum_{j=1,\neq 0}^{n}\beta_{j}Y_{j}^{b},X^{-}\geq X_{0},Y^{-g}\leq Y_{0}^{g},\;Y^{-b}\leq Y_{0}^{b};\;\;\sum_{j=1,\neq 0}^{n}\beta_{j}=1,Y^{-g}\geq 0,\beta \geq 0$$

In the above Equation (1), ρ* is the target efficiency value; *X* is the input; *Y^g^* is the desired output; *Y^b^* is the non-desired output; *X^−^* is the input slackness; *Y^−g^* is the slack in desired output; *Y^−b^* is the slackness for non-desired output; *β* is the weight vector; and model subscript “0” is the evaluated decision unit. ρ* is strictly monotonically decreasing with respect to Y^−g^, Y^−b^, X^−^, and 0 ≤ ρ* ≤ 1. For a specific decision unit, when ρ* = 1 and Y^−g^, Y^−b^, X^−^ are all equal to 0, it indicates that the decision cell is valid; conversely, if *ρ*^* < 1, it indicates that the decision unit is invalid and the corresponding input–output needs to be improved.

#### 3.1.2. Benchmark Regression Model

To examine the impact of *ER*s on *AEE*, the following benchmark regression model is constructed in this paper:

(2) $$AEE_{it}=\beta_{0}+\beta_{1}LnER_{it}+\beta_{2}ControlX_{it}+\sigma_{i}+\upsilon_{t}+\varepsilon_{i\;}$$

where *i* denotes the Chinese provinces, *t* denotes the year, *AEE_it_* indicates agriculture eco-efficiency, *LnER_it_* denotes the logarithm of environment regulation, *ControlX_it_* represents a series of control variables affecting *AEE*, *σ_i_* denotes the individual fixed effect, *υ_t_* denotes the time fixed effect, and *ε_i_* is the random error term. *β*_0_ is the intercept term, and *β*_1_ is the coefficient of the effect of *ER* on *AEE*.

### 3.2. Selection of Variables

Explained variables: agriculture eco-efficiency (*AEE*). Firstly, the indicators are selected in two dimensions of input and output of agriculture (planting industry), among which nine indicators are selected for input indicators, such as total agricultural machinery power (TAMP), the use of water in agriculture (IA), sown area (SA), agricultural labor force (ALF), agricultural electricity consumption (AEC), fertilizer (Fert), pesticide (Ptc), agricultural film (AF) and diesel (Ds). Outputs are divided into desired and undesired outputs, where the desired output is agricultural output (Agr-GDP), and there are two types of undesired outputs: fertilizer and film residues (FFR) and carbon emissions (CO_2_-E) from agricultural planting processes. Secondly, the residual coefficients of chemical fertilizers, pesticides and agricultural films in the indicators are based on the *First National Pollution Source Census of China*. For the calculation of carbon emissions generated in agricultural production, we refer to existing studies [41,42] and go by the following carbon emission factors: fertilizer 0.896 (kg/kg), pesticide 4.934 (kg/kg), agricultural film 5.180 (kg/kg), diesel 0.693 (kg/kg), tillage 20.476 (kg/hm^2^), and irrigation 312.60 (kg/hm^2^). Since the DEA method for solving production efficiency is independent of the data dimensionality, the data are not dimensionless in this paper. Please see Table A1 in Appendix A for the results of *AEE* measurement in China

Core explanatory variables: environmental regulation (*ER*) is one of the important variables analyzed in this study. Although *ER* is generally classified into three types: guidance, incentive, and constraint, according to relevant scholars, the measurement of each type of *ER* varies widely when it comes to different industry sectors or different research objectives. In terms of the construction of the formula, Fang Zeng et al. (2021) argued that the level of economic development can better reflect the intensity of local *ER*, and the area of the region can significantly influence the intensity of local *ER* [43]. Xuehui Wang and Guofeng Gu (2016) took two-thirds of the regional area as the intra-regional distance and used the regional area and circumference as key elements to construct the Euclidean linear distance of the region, which would provide a better geographical distance explanation for the implementation of *ER* at the regional scale [44]. Therefore, referring to Wang and Gu (2016) and Zeng et al. (2021), this paper uses the adjustment factor to improve the total GDP of regional economic development level to measure the intensity of *ER* [43,44]. In order to avoid the effect of heteroskedasticity in the regression due to *ER* units that are too large, this paper will treat the logarithm of *ER* with the following equation:

(3) $$LnER_{i}=Ln\left(GDP_{i}\times \frac{1}{\frac{2}{3}\times \sqrt{\frac{area_{i}}{\pi }}}\right)$$

*LnER_i_* denotes the logarithm of *ER* intensity in region *i*, *GDP_i_* denotes the gross regional product, *area_i_* is the area of each province’s administrative region, and *π* is the circumference.

Mediating variable: rural residents’ health investment (RRHI), measured by health care and medical services expenditures in rural residents’ living expenses (Medical), and logarithmic (Lnmedical).

Control variables: based on the studies of Baoyi Wang, Weiguo Zhang (2018) and Dai SH et al. (2021), for the reliability of data sources and the scientific nature of the study, the per capita sown area, industrial structure ratio, mechanization level and labor input per unit area were selected as control variables in this paper [28,42]. Among them, the sown area per capita (SAPC), is expressed by the ratio of primary industry employees to sown area. When the scale of agricultural production is small, farmers will put a lot of chemical fertilizers and pesticides on the limited arable land in order to obtain high yield and income. However, these production methods are relatively crude and their awareness of ecology and environmental protection is weak, thus leading to low *AEE*. When the scale of agricultural production is increased, various agricultural production materials inputs will increase, and additional employment costs will also be incurred. When farmers still maintain a crude production and operation method or adopt large fuel-based mechanized operations, they will also have some impact on *AEE*. The industrial structure (IS), expressed as the ratio of agricultural GDP to agricultural, forestry, and fishery GDP. The higher ratio of agricultural industry structure indicates that the development of the region’s economy relies more on the development of agricultural plantation industry; however, under the current situation of insufficient development of modern agriculture, family agricultural production is cruder and cannot achieve the scientific and fine management of agricultural production, and the *AEE* is low. The level of agricultural mechanization (LAM), expressed by the total power of agricultural machinery per unit area. The level of agricultural mechanization represents to a certain extent the level of agricultural modernization in a region, and its advantageous role in the agricultural production process is becoming increasingly clear. The improvement in the level of agricultural mechanization will improve land productivity and increase farmers’ income. However, the increase in the level of agricultural mechanization will increase the use of chemical fuels such as oil and diesel and pollution emissions, which creates some problems in terms of environmental pollution and could inhibit the improvement in *AEE*. Labor input per unit area (LI) is expressed as the ratio of primary industry employees to total crop sown area. The higher the quantity of labor input, the higher the degree of labor-intensive production, which relies mainly on labor input to achieve high output, indicates that the more rudimentary the production method, the lower the *AEE*. See Table 1 for specific information on all variables.

## 4. Regression Analysis

### 4.1. Benchmark Regression Analysis

In this paper, after the Hausman test, the *p*-value is 0.0001, which is less than 0.05, so the fixed-effects model is chosen for estimation, which includes an individual fixed effects and two-way fixed-effects model with the addition of control individuals and time until the parameter estimation of the regression coefficients of *ER* affecting *AEE*, Table 2 shows the regression results of individual fixed and two-way fixed-effects models, where Table 2, Model (1) and (2) are the regression results for fixed provinces, and (3) and (4) are the regression results for fixed time and provinces. The results show that before and after the addition of control variables, the regression results of both models show that *ER* has a significant positive effect on *AEE*, and R^2^ also increases with the addition of control variables; therefore, it can be proved that the level of *AEE* can be significantly increased with the effect of *ER*, and thus hypothesis H1 is proved. In the fourth column of Table 2, the regression results of the two-way fixed effects model with the addition of control variables are as follows: for every 1% unit increase in *ER*, the *AEE* will increase by 0.879%.

### 4.2. Endogeneity Problem

Although the benchmark regression results show a significant positive effect of *ER* on *AEE*, *ER* and *AEE* may have a two-way causal relationship, which leads to endogenous problems. *ER* can not only promote *AEE*; conversely, if the level of *AEE* is low, it means that the environmental quality in the agricultural production process is poor, which will lead the government to strengthen the management of the agricultural production environment and improve the strength of *ER*, so there is a two-way causality between *ER* and *AEE*. In this paper, we choose the IV-2SLS method and refer to the first-order lag of *ER* as an instrumental variable [45] for addressing the endogeneity issue. Table 3 shows the IV-2SLS regression results. In the first stage, the effect of *ER* first-order lag term of *ER* on *ER* is positive, with *p*-values significant at the 1% level, and the Kleibergen–Paap F-value in the first stage is much greater than 10, which is consistent with the rule of thumb (Model (5))/ The second stage regression results show that there is still a significant positive effect of *ER* on *AEE* (Model (6)).

### 4.3. Robustness Test

To ensure the reliability of the regression results, this paper further conducts robustness tests. Traditional robustness tests are generally performed by replacing core variables, changing the model, and increasing or decreasing the sample size. Based on the existing data collection and the simplicity of the model operation, this paper adopts the method of transforming the model and replacing the explanatory variables to conduct the robustness test.

The type of data in this paper is panel data, so the dynamic panel estimation method, GMM method, is used to perform the robustness test. The GMM estimation method is divided into two types of differential GMM and systematic GMM estimation, but Li Gu-Cheng et al. (2018) pointed out that systematic GMM estimation may cause the problem of over-identification of instrumental variables [46]; therefore, this paper chooses a differential GMM estimation method to verify the robustness of the model. The results of differential GMM estimation are shown in Model (7) of Table 4, which shows that *ER* still have a significant positive effect on *AEE*.

Second, this paper uses the inverse of the non-desired output of agricultural production (Nonoutput^−1^) instead of *AEE*. This is because a higher degree of *AEE* indicates a lower degree of non-desired agricultural output and a higher value of the inverse of non-desired output (*AEE* and non-desired output > 0). Therefore, this paper measures *AEE* by the inverse of agricultural non-desired output, and if *ER* can significantly promote *AEE*, its coefficient will be positive, and a non-desired output will be reduced. In Table 4, the coefficient of *ER* is still significantly positive, indicating that, as the intensity of *ER* increases, the non-desired output decreases, and the inverse of the non-desired output increases, thus promoting *AEE*. Model (8) of Table 4 shows that *ER* can reduce the agricultural non-desired output and promote *AEE*, while controlling for time and individual effects.

## 5. ER, RRHI, and AEE

### 5.1. Analysis of Mediating Effects

#### 5.1.1. Model Construction

In this paper, the mediating effect is tested through the following three steps. First, *ER* is regressed on the mediating variable RRHI, and if the regression coefficient is significantly positive, it indicates that *ER* can promote residents’ investment in their health. Second, the regression of *AEE* uses RRHI, and if the coefficient is significantly positive, it indicates that RRHI can significantly promote *AEE*. Finally, if the coefficients of *ER* and RRHI are included in the model at the same time, and the coefficient of *ER* is still significantly positive and RRHI is also significantly positive, it indicates that the variable *ER* affects *AEE* through the mediating variable, RRHI. This paper constructs a model based on the following:

(4) $$LnMedical_{it}=\alpha_{0}+\alpha_{1}LnER_{it}+\sum_{i=1}^{N}\beta_{i}ControlX_{it}+\sigma_{i}+\upsilon_{t}+\xi_{it}$$

(5) $$AEE_{it}=\delta_{0}+\delta_{1}LnMedical_{it}+\sum_{k=1}^{N}\kappa_{i}ControlX_{it}+\sigma_{it}+\upsilon_{it}+\tau_{it}$$

(6) $$AEE_{it}=\gamma_{0}+\gamma_{1}LnER_{it}+\gamma_{2}LnMedical_{it}+\sum_{\lambda =1}^{N}\lambda_{i}ControlX_{it}+\sigma_{it}+\upsilon_{it}+\mu_{it}\;$$

*LnMedical_it_* denotes the logarithm of RRHI; *ControlX_it_* is the control variable; $\beta_{i}$, $\kappa_{i}$*,* $\lambda_{i}$ are the coefficients of the control variables in each model, respectively; *ξ_it_*, *τ_it_*, *μ_it_*, are random errors term; *σ_i_* denotes individual fixed effects; and *υ_t_* denotes time fixed effects.

#### 5.1.2. Empirical Analysis

We must consider that *ER* may improve *AEE* by promoting rural residents to make RRHI and improve human capital, thus increasing *AEE*. The mediating variable in this paper is taken as RRHI, which is expressed as the logarithm of rural residents’ health care expenditure (*Lnmedical*), and the data are obtained from the China Rural Statistical Yearbook and provincial statistical yearbooks from 2010–2019.

The regression results of steps one to three are shown in Models (9) to (11) of Table 5. In Model (9) of Table 5, the regression coefficient of *ER* is positive at the 10% significance level, indicating that *ER* has a positive effect on RRHI, and H2 is verified. In Model (10), the regression result of RRHI is positive at the 5% significance level, indicating that as RRHI increases, it also significantly improves *AEE*, and H3 is verified. In Model (11), the regression results, including both *ER* and RRHI, show that the regression coefficients of both variables are significant and positive, indicating that RRHI plays a mediating role in the effect of *ER* on *AEE*, and H4 is verified.

In summary, this paper finds that *ER* can effectively enhance *AEE* and further improve *AEE* by promoting RRHI to achieve *AEE*.

### 5.2. Heterogeneity Analysis

This paper investigates the mechanisms of *ER*, RRHI, and *AEE* in 31 provinces of China; however, differences in economic development across regions may have an impact on the research in this paper. Therefore, this paper first divides China’s 31 provinces into the traditional three regions—eastern, central and western—according to the classification criteria of the National Development and Reform Commission, which represent high, middle, and low levels of economic development, and then analyzes the effect of *ER* on *AEE*; the regression results are shown in Table 6. The regression results in Model (12) and Model (14) of Table 6 show that there is a significant positive effect of *ER* on *AEE* in the eastern and western regions, and it passes the 1% significance level test. This indicates that *ER*s have a positive impact on *AEE* and green economic development at both high and low levels of economic development. This also illustrates the reality of agricultural production in the eastern and western regions of China. In the western regions with lower levels of economic development, such as Tibet Autonomous Region (Tibet), Xinjiang Uygur Autonomous Region (Xijiang), Qinghai, Inner Mongolia Autonomous Region (Neimenggu), and Sichuan, most farmers are still using traditional methods of agricultural production, and these regions do not improve *AEE* by sacrificing the environment and putting in large amounts of chemicals. On the contrary, in regions with a high level of economic development, such as Guangdong, Jiangsu, Zhejiang, and Shandong, where people’s income level determines that they are more concerned about health, *ER* will work to improve agricultural production methods and promote *AEE* to meet the demand for higher-quality food from society’s residents. The impact of *ER* on *AEE* in the central region is negative, and the *p*-value is not significant (Model (13)). The possible reason for this is that the central region requires a lot of chemical inputs to increase the productivity per unit of land during the stage of economic development from a low to a high level; the demand for agrochemicals is huge and their prices are cheaper at this stage, but this will cause some harm to the environment. At this stage, the cost to the government of increasing *ER* to improve *AEE* will become higher [47], to promote agricultural productivity and reduce government financial pressure, the government may deregulate farmers’ use of fertilizers and pesticides, but this also reduces the level of agroecological efficiency. Therefore, at this stage, increasing the level of *ER* will not effectively improve *AEE*, and forcing an increase in *ER* will result in the phenomenon of reduced efficiency of *AEE*.

## 6. Conclusions and Recommendations

This paper empirically analyzes the effect of *ER* on *AEE*, and the mediating effect of RRHI in *ER* and *AEE* using the panel data of 31 provinces in China from 2009 to 2018. It was found that, (1) regarding the role of *ER* on *AEE* in the univariate linear regression model, *ER* has a significant positive effect on *AEE*. After adding some control variables, the promotion effect of *ER* on *AEE* was significantly increased, suggesting that, at this stage, *ER* in China has a significant contribution to *AEE*. (2) The mediating effect model based on RRHI shows that RRHI can act as a mediating variable between *ER* and *AEE* better. That is, *ER* can increase the level of *AEE* by promoting RRHI. This suggests that environmental regulation positively contributes to both RRHI and *AEE*, and that RRHI can also improve *AEE*. (3) When China is divided into three regions—eastern, central and western—the heterogeneity results show that the impact of *ER* on *AEE* is greatest in the economically developed eastern region, followed by the western region, while the *ER*s in the central region have a negative (but not significant) impact on *AEE*. It also means that the rapid development of agriculture in the low-to-high development stage of regional or national economy is carried out at the expense of ecological environment, while at a certain level of socio-economic development, the Government and residents begin to gradually increase the ecological awareness and intensity of environmental regulations, and thus the agricultural development method begins to develop into a green economy. Therefore, the impact of *ER* on *AEE* is not always facilitated at different stages of economic development.

These findings have important implications for *ER* in promoting human health and *AEE* and provide a better realistic basis for how to formulate environmental regulation policy implementation under different stages of regional economic development. First of all, *ER* can promote *AEE*, so governments at all levels should pay attention to the role of *ER*, introduce policy documents related to the green development of agriculture, mobilize farmers’ enthusiasm for green production, and play the role of supervision by the public. Secondly, RRHI plays an intermediary role in *ER* and *AEE*; therefore, *ER* should be used to mobilize residents’ concerns and investments in the environment and their health. We should explore the green development model of “government guide, social supervision, and people participation”, and play the role of *ER* at multiple levels to raise residents’ concern for health. Finally, *ER*s have clear regional heterogeneity. Therefore, local governments should implement *ER*s according to the degree of socio-economic development, the cost of *ER*s and the cost of green agricultural production, and should not adopt a “one-size-fits-all” approach to promote green agricultural production. For example, in medium economic regions, *ER* should be moderately reduced, while in economically developed regions, *ER* should be increased, so that environmental regulations can play a role in promoting green agricultural production.

## 7. Limitations and Perspectives

This paper is of great significance for describing the mechanism of the role of *ER* in *AEE*, and it also provides help to different regional governments in formulating environ-mental policies; however, this paper also has the following limitations. (1) This study uses indicators, such as the level of economic development and size of administrative areas, to measure the level of environmental regulation, which can reflect the strength of environmental regulation to some extent but hardly represents the meaning of the three types of environmental regulation. (2) Although this article uses an accurate method to measure regional *AEE*, the result obtained using macro data may differ from field surveys.

Based on the limitations of this paper and the trend of related research, this paper proposes the next research intention and outlook: (1) By focusing on a typical region and using questionnaires to assess local *ER*, RRHI and *AEE* can better match more realistic measurements. (2) Currently, the economic development in some regions is still at the expense of the environment, and the local environmental quality and health level of residents still requires attention. Therefore, interested scholars can focus on such regions and study in more depth the evolution of local environmental policies and changes in the health level of residents as the regional economy develops, which have important implications for environmental protection and residents’ health.

## Figures and Tables

**Table 1:** Descriptive statistical analysis.

Variables	Variable Specific Definition	Mean	SD	Minimum	Maximum
Agricultural electricity consumption (AEC)	Agricultural electricity consumption	266.3	397.2	0.800	1933
Agricultural labor force (ALF)	Agriculture, forestry, animal husbandry and fishery employees × agriculture GDP/agriculture, forestry and fishing GDP	945.1	681.9	33.38	2765
Sown area (SA)	Total crop sown area	5292	3777	103.8	14,903
The use of water in agriculture (IA)	Irrigated area	2067	1611	109.7	6120
Total agricultural machinery power (TAMP)	Total mechanical power	3228	2923	94	13,353
Fertilizer (Fert)	Fertilizer input	186.9	148.1	4.700	716.1
Agricultural film (AF)	Agricultural film input	78,074	66,961	441	322,965
Diesel (Ds)	Diesel input	67.40	60.46	1.900	301.9
Pesticide (Ptc)	Pesticide input	55,971	43,650	921	169,043
Agricultural output (Agr-GDP)	Agricultural GDP	1600	1193	39.10	4974
Carbon emissions (CO_2_-E)	Carbon emissions from agricultural production processes	350.9	250.7	13.91	1049
Fertilizer and film residues (FFR)	Agricultural film and fertilizer residue	18,154	15,569	102.6	75,094
Agriculture eco-efficiency (*AEE*)	agro-ecological efficiency	0.6007	0.2311	0.23386	1.1512
Environment regulation (*LnER*)	Ln (regional GDP × (2/3(area of regional jurisdiction × 1/circumference)^1/2^)^−1^)	3.941	0.575	2.029	5.081
Industiral structure (IS)	Agriculture GDP/agriculture, forestry and fishery GDP	0.53	0.0881	0.302	0.899
The level of agricultural mechanization (LAM)	Total agricultural machinery power/total crop sown area	0.669	0.347	0.25	2.451
The sown area per capita (SAPC)	Total crop area sown/rural population	6.155	3.222	1.422	19.92
Unit area labor Inputs (LI)	Employees in the primary sector/total area sown to crops	0.203	0.102	0.050	0.703
Rural residents’ health investment (Lnmedical)	Ln (rural residents’ health care expenditure)	2.773	0.281	1.786	3.299

**Table 2:** Benchmark regression model analysis.

Variables	Model (1)	Model (2)	Model (3)	Model (4)
	*AEE*	*AEE*	*AEE*	*AEE*
*LnER*	0.95173 ***	0.990 ***	0.427 **	0.879 ***
	(21.69)	(23.01)	(2.18)	(4.39)
IS		1.267 ***		1.373 ***
		(6.96)		(7.07)
LAM		−0.289 ***		−0.244 ***
		(−5.27)		(−4.06)
SAPC		0.0299 ***		0.0256 **
		(3.25)		(2.53)
LI		1.491 ***		1.328 ***
		(5.80)		(4.66)
cons	−3.149	−4.267 ***	−1.193	−3.856 ***
	(−18.20)	(−22.03)	(−1.65)	(−4.97)
Times-fixed	NO	NO	YES	YES
Province-fixed	YES	YES	YES	YES
R^2^	0.6285	0.7268	0.6641	0.7441
*N*	310	310	310	310

Note: t-statistics in parentheses, ** *p* < 0.05, *** *p* < 0.01.

**Table 3:** 2 SLS Regression.

Variables	Model (5) *AEE*	Model (6) *AEE*
*L.LnER*	0.8918 ***	-
	(0.088)	
*LnER*	-	1.042 ***
		(0.0568)
IS	−0.0408	1.325 ***
	(0.029)	(0.2632)
LAM	−0.015	−0.2483 **
	(0.0136)	(0.0823)
SAPC	−0.0016	0.0286 ***
	(0.0015)	(0.0121)
LI	0.072	1.453 ***
	(0.033)	(0.2901)
*N*	279	279
Underidentification test (Kleibergen-Paaprk LM statistic)	97.88, *p* = 0.0000	
Weak identification test (Cragg–Donald Wald F statistic):	12,695.96	
(Kleibergen–Paap rk Wald statistic):	10,259.08	

Note: Robust standard error in parentheses, ** *p* < 0.05, *** *p* < 0.01.

**Table 4:** Robustness Test.

Variables	Model (7)	Model (8)
	*AEE*	Nonoutput^−1^
*L.AEE*	0.128	-
	(1.64)	-
*LnER*	0.920 ***	0.00626 **
	(11.26)	(2.06)
IS	1.060 ***	−0.0016
	(6.20)	(−0.53)
SAPC	−0.302 ***	0.00018
	(−5.19)	(0.35)
LAM	0.0246 **	0.00006
	(2.09)	(0.63)
LI	1.683 ***	−0.0012 ***
	(4.94)	(−0.48)
cons	−3.951 ***	−0.0221 ***
	(−12.80)	(−3.05)
Times-fixed	YES	YES
Province-fixed	YES	YES
Sargan test	0.819	-
AR (1)	0.0369	-
R^2^	-	0.226
*N*	279	310

Note: The numbers in parentheses in Model (7) of the table are z-statistics, and the numbers in parentheses in Model (8) are t-statistics; ** *p* < 0.05, *** *p* < 0.01.

**Table 5:** Mediating effect test.

Variables	Model (9)	Model (10)	Model (11)
	*Lnmedical*	*AEE*	*AEE*
*Lnmedical*	-	0.172 **	0.133 *
		(2.13)	(1.69)
*LnER*	0.297 *	-	0.839 ***
	(1.91)		(4.17)
IS	0.216	1.401 ***	1.344 ***
	(1.43)	(7.01)	(6.91)
LAM	−0.0524	−0.137 **	−0.237 ***
	(−1.12)	(−2.42)	(−3.95)
SAPC	0.000853	0.0101	0.0255 **
	(0.11)	(1.05)	(2.53)
LI	−0.495 **	0.969 ***	1.394 ***
	(−2.24)	(3.51)	(4.87)
cons	1.414 **	0.945 ***	−4.04 ***
	(2.34)	(−3.78)	(−5.18)
R^2^	0.9057	0.7302	0.7469
Times-fixed	YES	YES	YES
Province-fixed	YES	YES	YES
*N*	310	310	310

Note: t-statistics in parentheses, * *p* < 0.10, ** *p* < 0.05, *** *p* < 0.01.

**Table 6:** Analysis of heterogeneity regression results.

Variables	Model (12)	Model (13)	Model (14)
	*AEE*	*AEE*	*AEE*
*LnER*	1.424 ***	−0.189	1.285 ***
	(3.12)	(−0.54)	(3.46)
CYJG	0.928 ***	1.938 ***	1.145 ***
	(2.67)	(6.54)	(3.47)
LAM	−0.1912	0.196 **	−0.556 ***
	(−1.52)	(2.30)	(−5.85)
SAPC	0.0582 ***	−0.017	0.0883 **
	(3.43)	(−1.01)	(2.50)
LI	1.305 ***	0.89	1.138 *
	(3.24)	(1.06)	(1.83)
cons	−6.546 **	−0.105	−4.772 ***
	(−3.38)	(−0.07)	(−3.93)
R^2^	0.864	0.8159	0.7789
Times-fixed	YES	YES	YES
Province-fixed	YES	YES	YES
*N*	90	110	110

Note: t-statistics in parentheses, * *p* < 0.10, ** *p* < 0.05, *** *p* < 0.01.

## Data Availability

Publicly available datasets were analyzed in this study. This data can be found here: (https://data.stats.gov.cn/easyquery.htm?cn=E0103, accessed on 10 September 2021), and partial data were obtained from the *China Rural Statistical Yearbook*, purchased at the author’s expense, and are available from the first author (K.Z.) upon request.

## Appendix A

This paper focuses on the analysis of the role of *ER* and RRHI in ARR. A single agricultural eco-efficiency result is not used as the main description object in this paper; if the reader wants to observe this, please see Appendix A in Table A1.

**Table A1:** Agricultural eco-efficiency value.

Year	City	*AEE*	City	*AEE*	City	*AEE*	City	*AEE*	City	*AEE*	City	*AEE*	City	*AEE*
2009	Anhui	0.2400	Beijing	0.6212	Chongqing	0.3093	Fujian	0.4146	Gansu	0.2339	Guangdong	0.3693	Guangxi	0.4694
2010	Anhui	0.2766	Beijing	0.6733	Chongqing	0.3469	Fujian	0.4898	Gansu	0.2827	Guangdong	0.4169	Guangxi	0.5406
2011	Anhui	0.2993	Beijing	0.7297	Chongqing	0.4027	Fujian	0.5642	Gansu	0.3018	Guangdong	0.4855	Guangxi	0.6207
2012	Anhui	0.3179	Beijing	0.8089	Chongqing	0.4467	Fujian	0.6179	Gansu	0.3410	Guangdong	0.5088	Guangxi	0.6833
2013	Anhui	0.3391	Beijing	0.8979	Chongqing	0.4701	Fujian	0.6778	Gansu	0.3736	Guangdong	0.5445	Guangxi	0.7482
2014	Anhui	0.3595	Beijing	0.8861	Chongqing	0.4912	Fujian	0.7595	Gansu	0.3882	Guangdong	0.5691	Guangxi	0.7935
2015	Anhui	0.3510	Beijing	1.0039	Chongqing	0.4805	Fujian	0.6614	Gansu	0.3089	Guangdong	0.6010	Guangxi	0.7428
2016	Anhui	0.3795	Beijing	1.0159	Chongqing	0.5846	Fujian	1.0442	Gansu	0.4549	Guangdong	0.6621	Guangxi	1.0329
2017	Anhui	0.3873	Beijing	1.0132	Chongqing	0.5877	Fujian	0.8613	Gansu	0.3891	Guangdong	0.7594	Guangxi	0.9137
2018	Anhui	0.3893	Beijing	1.1030	Chongqing	0.6472	Fujian	1.0011	Gansu	0.4296	Guangdong	1.0010	Guangxi	1.0351
2009	Guizhou	0.2863	Hebei	0.2967	Henan	0.3412	Heilongjiang	0.2942	Hainan	0.4901	Hubei	0.3362	Hunan	0.3382
2010	Guizhou	0.3164	Hebei	0.3844	Henan	0.4469	Heilongjiang	0.3176	Hainan	0.5169	Hubei	0.4227	Hunan	0.4096
2011	Guizhou	0.3091	Hebei	0.4461	Henan	0.4493	Heilongjiang	0.4109	Hainan	0.5590	Hubei	0.4932	Hunan	0.4810
2012	Guizhou	0.4021	Hebei	0.5146	Henan	0.5060	Heilongjiang	0.5445	Hainan	0.6090	Hubei	0.5525	Hunan	0.5233
2013	Guizhou	0.4497	Hebei	0.5948	Henan	0.5373	Heilongjiang	0.6916	Hainan	0.6399	Hubei	0.5655	Hunan	0.5796
2014	Guizhou	0.5760	Hebei	0.5881	Henan	0.5892	Heilongjiang	0.7370	Hainan	0.7216	Hubei	0.6059	Hunan	0.5987
2015	Guizhou	0.7529	Hebei	0.4494	Henan	0.5922	Heilongjiang	0.7947	Hainan	0.7511	Hubei	0.4405	Hunan	0.5737
2016	Guizhou	0.8215	Hebei	0.6269	Henan	0.6156	Heilongjiang	0.6777	Hainan	1.0055	Hubei	0.7318	Hunan	0.6826
2017	Guizhou	0.8967	Hebei	0.4988	Henan	0.6232	Heilongjiang	0.9133	Hainan	1.0262	Hubei	0.5255	Hunan	0.7180
2018	Guizhou	1.1513	Hebei	0.5740	Henan	0.7319	Heilongjiang	1.0607	Hainan	1.1096	Hubei	0.5504	Hunan	0.7899
2009	Jilin	0.2982	Jiangsu	0.3953	Jiangxi	0.2440	Liaoning	0.3214	Neimenggu	0.2579	Ningxia	0.3299	Qinghai	0.4870
2010	Jilin	0.3263	Jiangsu	0.4655	Jiangxi	0.2598	Liaoning	0.3825	Neimenggu	0.2991	Ningxia	0.4215	Qinghai	0.5758
2011	Jilin	0.3671	Jiangsu	0.5879	Jiangxi	0.2911	Liaoning	0.4286	Neimenggu	0.3486	Ningxia	0.4369	Qinghai	0.5620
2012	Jilin	0.4242	Jiangsu	0.6997	Jiangxi	0.3110	Liaoning	0.5167	Neimenggu	0.3659	Ningxia	0.4595	Qinghai	0.7784
2013	Jilin	0.4574	Jiangsu	0.7637	Jiangxi	0.3707	Liaoning	0.5790	Neimenggu	0.4168	Ningxia	0.5087	Qinghai	0.7817
2014	Jilin	0.4914	Jiangsu	0.8269	Jiangxi	0.3921	Liaoning	0.6019	Neimenggu	0.4378	Ningxia	0.5407	Qinghai	1.0036
2015	Jilin	0.3813	Jiangsu	0.9393	Jiangxi	0.4616	Liaoning	0.6292	Neimenggu	0.4576	Ningxia	0.5873	Qinghai	0.7799
2016	Jilin	0.4345	Jiangsu	0.9623	Jiangxi	0.4972	Liaoning	0.6835	Neimenggu	0.4200	Ningxia	0.6560	Qinghai	0.8399
2017	Jilin	0.3076	Jiangsu	1.0035	Jiangxi	0.5116	Liaoning	0.5508	Neimenggu	0.4243	Ningxia	0.6581	Qinghai	0.9003
2018	Jilin	0.3488	Jiangsu	1.0135	Jiangxi	0.5510	Liaoning	0.6296	Neimenggu	0.4733	Ningxia	1.0195	Qinghai	1.0226
2009	Sichuan	0.3629	Shandong	0.3816	Shanghai	0.7482	Shanxi	0.2561	Shaanxi	0.3683	Tianjin	0.4153	Xinjiang	0.3701
2010	Sichuan	0.4054	Shandong	0.4393	Shanghai	0.8272	Shanxi	0.2976	Shaanxi	0.4760	Tianjin	0.5124	Xinjiang	0.6440
2011	Sichuan	0.4870	Shandong	0.4654	Shanghai	1.0191	Shanxi	0.3286	Shaanxi	0.5739	Tianjin	0.5465	Xinjiang	0.6241
2012	Sichuan	0.5719	Shandong	0.4893	Shanghai	1.0055	Shanxi	0.3532	Shaanxi	0.6310	Tianjin	0.6129	Xinjiang	0.7077
2013	Sichuan	0.6089	Shandong	0.5880	Shanghai	1.0055	Shanxi	0.3826	Shaanxi	0.7100	Tianjin	0.7072	Xinjiang	0.7326
2014	Sichuan	0.6566	Shandong	0.6503	Shanghai	1.0116	Shanxi	0.3973	Shaanxi	0.7938	Tianjin	0.7855	Xinjiang	0.7128
2015	Sichuan	0.7203	Shandong	0.6281	Shanghai	0.9685	Shanxi	0.3410	Shaanxi	0.7807	Tianjin	0.6200	Xinjiang	0.7446
2016	Sichuan	0.8454	Shandong	0.6444	Shanghai	0.8624	Shanxi	0.4222	Shaanxi	0.8787	Tianjin	1.0180	Xinjiang	0.7749
2017	Sichuan	0.9383	Shandong	0.6067	Shanghai	0.8989	Shanxi	0.3999	Shaanxi	0.9128	Tianjin	0.7470	Xinjiang	0.8694
2018	Sichuan	1.0666	Shandong	0.7218	Shanghai	1.0875	Shanxi	0.4190	Shaanxi	1.0782	Tianjin	1.1089	Xinjiang	1.0665
2009	Tibet	1.0608	Yunnan	0.2409	Zhejiang	0.3755								
2010	Tibet	0.9996	Yunnan	0.2402	Zhejiang	0.4540								
2011	Tibet	0.9986	Yunnan	0.2776	Zhejiang	0.5085								
2012	Tibet	1.0287	Yunnan	0.3291	Zhejiang	0.5508								
2013	Tibet	0.7977	Yunnan	0.3735	Zhejiang	0.6140								
2014	Tibet	0.8106	Yunnan	0.4023	Zhejiang	0.6525								
2015	Tibet	0.7639	Yunnan	0.3918	Zhejiang	0.6577								
2016	Tibet	0.5616	Yunnan	0.4226	Zhejiang	0.9094								
2017	Tibet	0.8866	Yunnan	0.4276	Zhejiang	0.9169								
2018	Tibet	1.0982	Yunnan	0.5483	Zhejiang	1.0276								
